# Methanolic extract of *Celosia argentea* var. crista leaves modulates glucose homeostasis and abates oxidative hepatic injury in diabetic rats

**DOI:** 10.1007/s00580-018-2702-9

**Published:** 2018-04-09

**Authors:** R. U. Hamzah, A. R. Lawal, F. M. Madaki, O. L. Erukainure

**Affiliations:** 1 Department of Biochemistry, Federal University of Technology, P.M.B. 65, Minna, Niger State Nigeria; 2 Nutrition and Toxicology Division, Federal Institute of Industrial Research, Oshodi, P.M.B. 21023, Lagos, Ikeja Nigeria

**Keywords:** *Celosia argentea*, Alloxan-induced diabetes, Free radicals, Hyperglycemia

## Abstract

*Celosia argentea* commonly known as cockscomb plant is widely used in folkloric medicine in the treatment and management of diabetes mellitus. The effect of methanolic extract of *Celosia argentea* var. *cristata L*. (CAVCL) leaves on blood glucose level, superoxide dismutase (SOD), catalase (CAT), alanine aminotransferase (ALT), aspartate aminotransferase (AST), alkaline phosphatase (ALP) activities, and malondialdehyde (MDA) level were evaluated in diabetic rats. Five groups of male albino rats consisting of 5 animals each were used for the present study. They were grouped as normal control, diabetic control, diabetic administered with 250 and 750 mg/kg b.w *C. argentea*, and 5 mg/kg b.w glibenclamide. Diabetes was induced with alloxan monohydrate intraperitoneally at 120 mg/kg b.w. The control and diabetic groups were given distilled water and rat chow for 21 days. Blood glucose level of each group was estimated every week, and at the end of the experiment, SOD, CAT, MDA and serum ALP, and AST and ALT activities were assayed in the liver and serum respectively of the experimental animals. The results showed a significant increase (*p* < 0.05) in serum AST, ALP, and ALT activities and reduction in SOD and CAT activities compared with normal control groups. The extract at both doses significantly lowered the high levels of the serum enzymes and increased the level of CAT and SOD. These results indicate an anti-hyperglycemia and antioxidative protective effect of *C. argentea* leaves.

## Introduction

Diabetes mellitus (DM) is a chronic metabolic disorder characterized by chronic hyperglycemia with impaired carbohydrate, protein, and fatty acid metabolisms arising from deficiencies in insulin secretion, action, or both (WHO 1999). It is characterized by increased hyperglycemia resulting from defective production or action of insulin, leading to several complications such as cardiovascular, renal, neurological, and ocular disorders (Erukainure et al. 2013). Growing evidence suggests that oxidative stress resulting from increased hyperglycemia plays a significant effect in the pathogenesis of DM and their complications (Brownlee 2001). In addition, antioxidant mechanisms which may alleviate oxidative stress are diminished in diabetic patients. Antioxidants are known to be significant in the treatment and control of DM and its complications. A diversity of local herbs and vegetables consumed by man is known to contribute significantly to the enhancement of human health either in prevention and/or cure of diseases because plants have for a long time served as a useful and natural source of therapeutic agents (Roberts and Tyler 1999). This has been attributed to their phytochemical and nutritional constituents. Among such plants is *Celosia argentea* var. *Cristata L.* (CAVCL).

*C. argentea* (family: Amaranthaceae) is a broad leafy vegetable commonly known as quail grass, feather cockscomb, and Lagos spinach. It is known locally as *Soko Yokoto* by the Yoruba-speaking ethnic group of Nigeria. It is known to grow very well in temperate and tropical regions (Yarger 2007). It is widespread through northern South America, the West Indies, tropical Africa, and tropical Asia where it matures as a wild flower. In the traditional medical practice, the decoctions of *C. argentea* seeds have been shown to be useful in the treatment of many diseases including jaundice, inflammation, healing of wounds, injuries, and diabetes mellitus Shah et al. 1993). Alcoholic extracts of the roots and seeds have been reported to possess antipyretic, anticancer, antidiabetic, antibacterial, diuretic, and antispasmodic properties (Vetrichelvan et al. 2002).

Although *C. argentea* decoction is demonstrated in folkloric medicine in the treatment of diabetes, there are, however, few scientific studies to evaluate its antidiabetic property. This study, therefore, is designed to further validate the hypoglycemic and antioxidant properties of this plant.

## Materials and method

### Reagent and chemicals

Trichloroacetic acetic acid (Sigma, London), thiobarbituric acid (Sigma, London), potassium chloride, (BDH Chemical ltd. England), Alloxan monohydrate (Sigma Chemical Co. St. Louis, MO, USA), Glibenclamide (Dana Pharmaceutical Company, Lagos), Randox Diagnostic kits for alanine amino transferase (ALT), aspartate aminotransferase (AST), Techo Diagnostic kit for Alkaline phosphatase (ALP).

### Plant materials

The plant was purchased from Ile-Epo market, Lagos, Nigeria, and was authenticated at Department of Botany, University of Lagos, Lagos, Nigeria. A voucher number LUH5435 was assigned and deposited in the herbarium.

The leaves were dried under room temperature and pulverized using an electric blender.

### Plant extraction

A portion of the powder 50 g was extracted with 300 ml of methanol. The extract was filtered using muslin cloth and Whatman’s no. 1 filter paper (Maidstone, UK) and concentrated in vacuo.

### Experimental animals

Male and female albino rats weighing 130–180 g were used for this study. The rats were obtained from Zoology Department, University of Ilorin, Ilorin, Nigeria. They were nourished ad libitum on feed manufactured by top feeds, Minna, and were acclimatized for 2 weeks in the Biochemistry Department Laboratory of Federal University of Technology, Minna, Nigeria. All animals were fasted overnight before commencement of the experiment.

These studies were carried out under the approval and guidelines of the ethical committee of the Federal University of Technology, Minna, Nigeria, in agreement to the Declaration of Helsinki.

### Acute toxicity and behavioral pattern studies

Acute toxicity study was carried out according to Organization for Economic Co-operation and Development (OECD 2009) guideline (OECD, 423). Thirty albino rats were grouped into six consisting of five animals each. The animals were fasted overnight (i.e., away from food only; water was not withdrawn) prior to dosing. Groups I–III received a single dose of 10, 100, and 1000 mg/kg b.w of the extract respectively, while groups IV–VI received 1600, 2000, and 5000 mg/kg respectively.

Animals were observed individually for the first 30 min with special attention being given in the first 4 h within 24 h and daily, thereafter, for the next 14 days for general behavioral, physiological, and pharmacological changes as well as lethality.

### Induction of diabetes

Experimental diabetes was induced in 20 out of 25 male albino rats by intraperitoneal injection of 120 mg/kg b.w alloxan monohydrate (Sigma Chemical Co. St. Louis, MO, USA) dissolved in normal saline. Diabetes was established by glucose oxidase method using the glucometer (Accu-check®) 72 h after alloxan injection. Rats with blood glucose level ≥ 200 mg/dl were isolated and used for this study.

### Experimental design

The rats were randomly allocated into five groups consisting of five rats each:

- Group A: were given distilled water and served as the control
- Group B: were diabetic and treated with 250 mg/kg b.w methanolic extract of *C. argentea*
- Group C: diabetic and treated with 750 mg/kg b.w methanolic extract of *C. argentea*
- Group D: diabetic and treated with 5 mg/kg b.w. Glibenclamide (standard drug)
- Group E: diabetic and received distilled water (Diabetic control)

Blood glucose was estimated once every week using a glucometer (ACCU-CHECK®). Administration of treatment was for a period of 3 weeks. At the end of the experimental period, they were fasted for 12 h and blood was collected by cardiac puncture under light anesthesia.

Serum was collected after centrifugation of blood samples at 2000 rpm for 10 min using bench centrifuge.

The excised liver of each animal was rinsed in ice cold 1.15% KCL solution, blotted and weighed. Homogenization of weighed liver was done with 0.1 M phosphate buffer (pH 7.2) and the resulting homogenate was centrifuged at 2500 rmp for 15 min at 4 °C. The supernatants were decanted and stored at − 20 °C until analysis.

### Serum hepatic biomarkers

Serum ALT and AST levels were determined using enzyme diagnostic kits, colorimetric method obtained from Randox (Reitman and Frankel 1957). Serum ALP activity was assayed using colorimetric endpoint method (1976) using Teco Diagnostic kits.

### Antioxidative activity

The homogenized hepatic tissues were assayed for oxidative stress marker enzymes vis-à-vis superoxide dismutase (SOD) (Misra and Fridovich 1972) and catalase activities (Sinha 1972).

Lipid peroxidation was analyzed by measuring the malondialdehyde (MDA) level of the hepatic tissue as described by Buege and Aust (1978).

### Statistical analysis

All values were expressed as mean ± SD of three animals per group. Statistical analysis was performed using one-way analysis of variance (ANOVA) and individual comparisons of the group mean values were done using Duncan’s test.

## Results and discussion

The cost of treating DM remains a bane in most developing countries, owing to their low economy status. This has led to increased rate of morbidity and mortality, thus the search for cheap affordable drugs with few or no side effects. In this study, we investigated the anti-hyperglycemic effect of the methanolic extract of *C. argentea* leaves vis-à-vis antioxidative and hepatic protective effects.

The test animals showed no significant signs of toxicity and changes in behavior before and after the administration of an oral dose of the methanolic extract of CAVCL leave as shown in Table 1. No mortality was recorded even at the highest dose, thus signifying that the extract has no toxic effect in rats.

**Table 1:** Acute toxicity effect of methanolic extract of *Celosia argentea* var. *cristata L.* leaves

Dose level (mg/kg)	Parameters monitored	Cage side observation	Mortality
10	Condition of the fur/skin	Normal	NIL
100	Eye dullness and ptosis	NIL	NIL
1000	Breathing abnormality	NIL	NIL
1600	Color and consistency of feces	Normal	NIL
2000	Gait	Normal	NIL
5000	Abdominal distension	NIL	NIL

Induction of diabetes led to significant increase (*p* < 0.05) in blood glucose level of 250.33 ± 6.65 mg/dL compared to the normal control group as depicted in Table 2. Treatment with glibenclamide, *C. argentea* extracts at 250 and 750 mg/kg b.w. resulted to a significant reduction to 104.33 ± 10.40, 103.33 ± 17.47, and 85.00 ± 5.19 mg/dL respectively (Table 2). This result compares favorably with the work of Edoga et al. (2013), which reported that the aqueous extract of *Moringa oleifera* leaves do possess a significant hypoglycemic activity in normo-glycemic and alloxan-induced diabetic rats in a dose-dependent manner and was almost comparable to the standard drug. Although no mechanism of action of *C. argentea* extract on blood glucose has been proposed, its mode of action may be due to presence of active compounds which imitate insulin action in the same manners as sulphonylureas that stimulate insulin secretion via closure of K^+^-ATP channels and stimulation of Ca^2+^ influx (Latha and Pari 2003).

**Table 2:** Effect of methanolic extract of *Celosia argentea* var. *cristata L.* leaves in alloxan induced diabetic rats

Treatment groups	Initial blood glucose	Blood glucose at day seven	Blood glucose at day fourteen	Blood glucose at day twenty one
A	83.66 ± 4.16^a^	81.00 ± 2.64^a^	86.66 ± 5.68^a^	85.00 ± 9.53^a^
B	457.00 ± 8.88^b^	172.00 ± 9.84^c^	235.67 ± 7.67^b^	103.33 ± 17.47^a^
C	487.00 ± 25.98^b^	133.66 ± 11.15^b^	248.33 ± 9.50^b^	85.00 ± 5.19^a^
D	555.67 ± 33.40^c^	171.35 ± 8.62^c^	328.00 ± 23.30^c^	104.33 ± 10.40^c^
E	468.00 ± 46.22^b^	252.33 ± 7.76^b^	254.00 ± 7.00^b^	250.33 ± 6.65^b^

Values with superscript different from the normal control are significantly different (*p* < 0.05). Values are mean of 3 determinations ± SD.

Keys: A = normal control group, B = diabetic +250 mg/kg b.w CAVCL, C = Diabetic +750 mg/kg b.w CAVCL, D = Diabetic + Glibenclamide 5 mg/kg b.w, E = Diabetic control

**Fig. 1:**
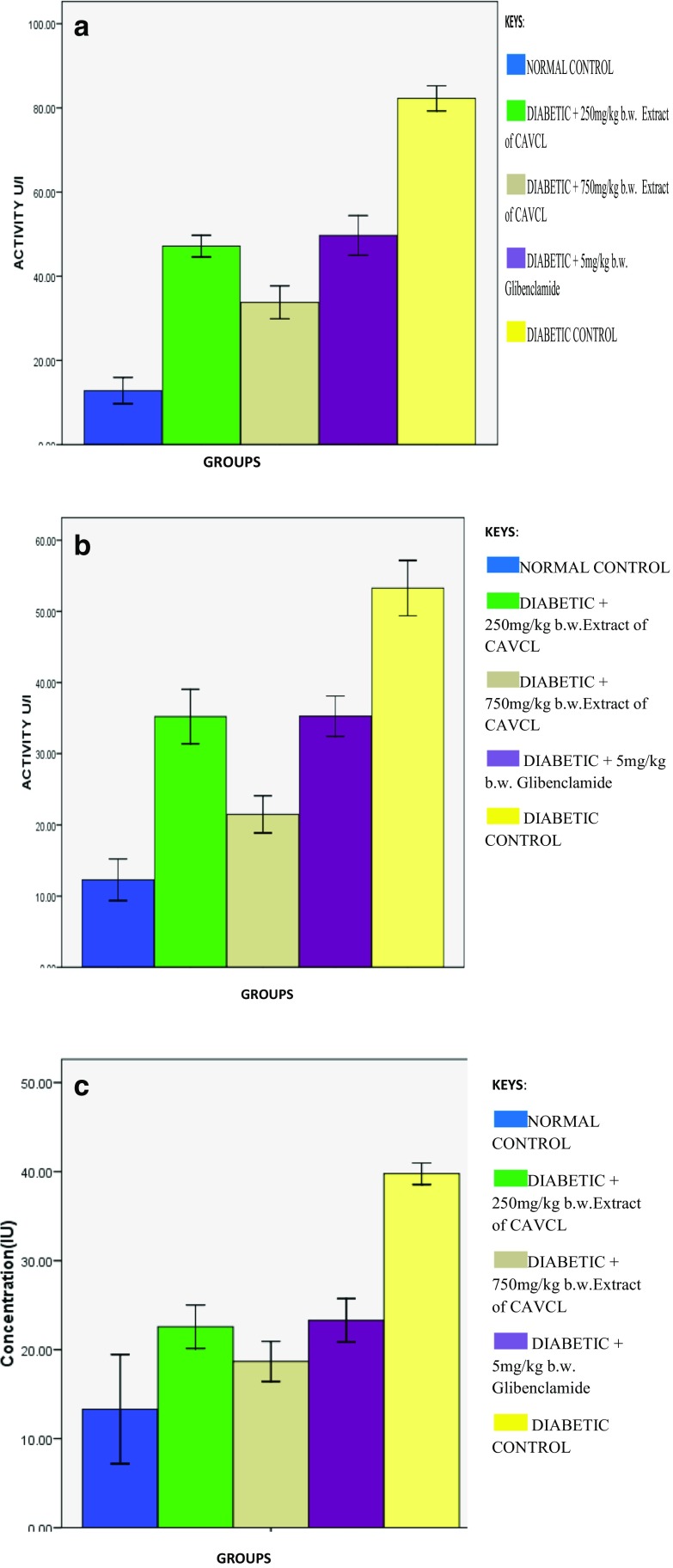
**a** Effect of methanolic extract of CAVCL on serum alanine transaminase concentration in alloxan-induced diabetic rats. **b** Effect of methanolic extract of CAVCL on serum aspartate transaminase concentration in alloxan-induced diabetic rats. **c** Effect of methanolic extract of CAVCL on alkaline phosphatase concentration in alloxan-induced diabetic rats

Serum levels of ALT, AST, and ALP were significantly (*p* < 0.05) high in diabetic control group compared with the normal group. The increased serum levels of these enzymes indicate an occurrence of hepatic injury (Erukainure et al. 2015). They are released into the blood stream due to inflammation of the liver. Administration of *C. argentea* extract at both doses and glibenclamide was able to reduce appreciably the elevated level of ALT (Fig. 1a). Significant reduction in aspartate transaminase level was observed for the groups administered with 250 mg/kg b.w *C. argentea* extract and glibenclamide. While serum ALP levels were reduced to near normal as shown in Fig. 1c, these reduced levels correspond to previous reports of Suneetha and Susatha (2010) who reported that the stem juice of *Musa paradisiaca* was able to significantly reduce the elevated levels of serum hepatic biomarkers in alloxan-induced diabetic rats after 21-day treatment in a dose-dependent manner. These results thus suggest a protective effect of the extract against hepatic injury.

**Fig. 2:**
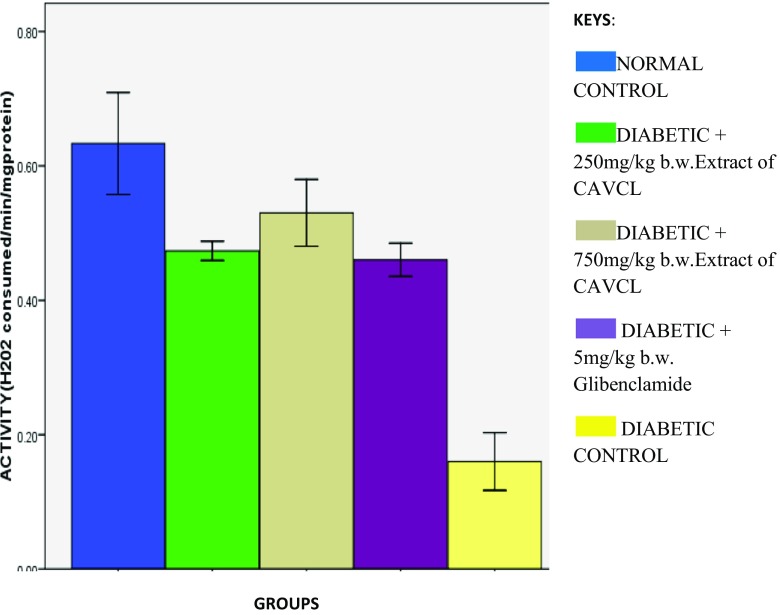
Effect of methanolic extract of CAVCL on catalase activities in alloxan-induced diabetic rats

**Fig. 3:**
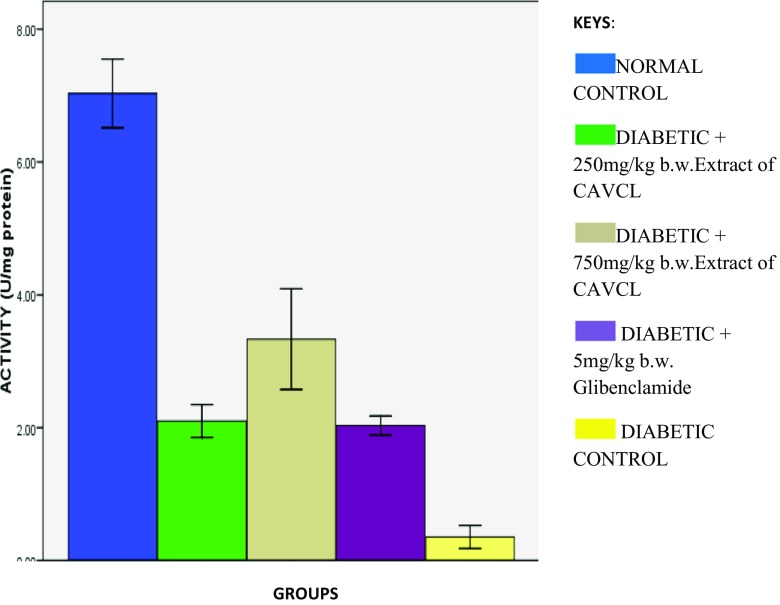
Effect of methanolic extract of CAVCL on superoxide dismutase activities in alloxan-induced diabetic rats

Induction of diabetes led to significant (*p* < 0.05) decrease in catalase and SOD activities as depicted in Figs. 2 and 3 respectively. These were significantly (*p* < 0.05) reversed on treatment with the extract and glibenclamide. Both SOD and catalase are major free radical-scavenging enzymes (Manonmani 2004). Their activities are usually little in diabetes mellitus (Vucic et al. 1997). The increased activities correspond to previous reports by Hamzah et al. (2012) on increased serum hepatic biomarkers in *Peperomia pellucida*-treated diabetic rats. The reduced SOD activity on induction of diabetes can be attributed to the unswerving harmful effect of free radicals on the enzyme or the diabetogenic agent alloxan. Previous studies have revealed the presence of an appreciable amount of iron, copper, and zinc in *C. argentea* plant (Chionyedua et al. 2009). Therefore, ability of this plant to increase catalase and superoxide dismutase activities can be attributed to the induction of the enzyme by these micronutrients as iron and Cu/Zn are metal cofactors for CAT and SOD respectively.

**Fig. 4:**
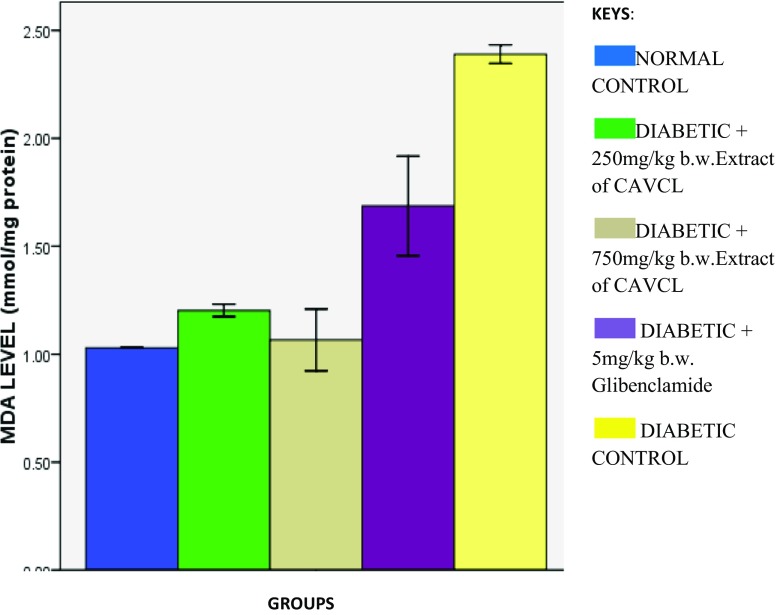
Effect of methanolic extract of CAVCL on lipid peroxidation in alloxan-induced diabetic rats

There was significant (*p* < 0.05) increase in MDA level on induction of diabetes, indicating an occurrence of lipid peroxidation as shown in Fig. 4. The increased MDA level can be attributed to the reduced SOD and catalase activities (Figs. 2 and 3) and may be responsible for the increased serum levels of hepatic biomarkers (Fig. 1a–c) due to leakage owing to peroxidation of hepatic membrane lipid. *C. argentea* extract at 750 mg/kg b.w. was effective in reducing the level of MDA significantly (*p* < 0.05) compared with the other treated groups. This corroborates the reduced serum hepatic biomarkers (Fig. 1a–c) and increased antioxidant activities (Figs. 2 and 3) after treatment with *C. argentea*.

## Conclusion

These results indicate an anti-hyperglycemia and antioxidative protective effect of *C. argentea* leaves, thus giving credence to its folkloric use in the treatment and management of diabetes. However, further studies are required to elucidate the molecular mechanism of action as well as secondary metabolites that may be responsible for these activities.
